# Prodomain–growth factor swapping in the structure of pro-TGF-β1

**DOI:** 10.1074/jbc.M117.809657

**Published:** 2017-11-05

**Authors:** Bo Zhao, Shutong Xu, Xianchi Dong, Chafen Lu, Timothy A. Springer

**Affiliations:** From the ‡ Children's Hospital Boston and Department of Biological Chemistry and Molecular Pharmacology, Harvard Medical School, Boston, Massachusetts 02115 and; the § Department of Medical Oncology and Department of Cancer Biology, Dana-Farber Cancer Institute, Harvard Medical School, Boston, Massachusetts 02215

**Keywords:** activin, bone morphogenetic protein (BMP), crystal structure, dimerization, transforming growth factor beta (TGF-β), heterodimer, prodomain, proprotein convertase, swapping, Arg-Gly-Asp-Leu-any-any-Leu/Ile (RGDLXX(L/I)), latent TGF-β-binding proteins (LTBPs), glycoprotein-A repetitions predominant protein (GARP), latency-associated peptide (LAP), growth factor (GF), Protein Data Bank (PDB)

## Abstract

TGF-β is synthesized as a proprotein that dimerizes in the endoplasmic reticulum. After processing in the Golgi to cleave the N-terminal prodomain from the C-terminal growth factor (GF) domain in each monomer, pro-TGF-β is secreted and stored in latent complexes. It is unclear which prodomain and GF monomer are linked before proprotein convertase cleavage and how much conformational change occurs following cleavage. We have determined a structure of pro-TGF-β1 with the proprotein convertase cleavage site mutated to mimic the structure of the TGF-β1 proprotein. Structure, mutation, and model building demonstrate that the prodomain arm domain in one monomer is linked to the GF that interacts with the arm domain in the other monomer in the dimeric structure (*i.e.* the prodomain arm domain and GF domain in each monomer are swapped). Swapping has important implications for the mechanism of biosynthesis in the TGF-β family and is relevant to the mechanism for preferential formation of heterodimers over homodimers for some members of the TGF-β family. Our structure, together with two previous ones, also provides insights into which regions of the prodomain–GF complex are highly structurally conserved and which are perturbed by crystal lattice contacts.

## Introduction

The 33 members of the TGF-β family include bone morphogenetic proteins, growth and differentiation factors, activins, and inhibins. They regulate all aspects of embryogenesis, major organ development, and homeostasis (1, 2). TGF-β1, -β2, and -β3 regulate development, cell fate, wound healing, and immune responses. TGF-β protein monomers are biosynthesized with an N-terminal prodomain of ∼250 residues and a C-terminal growth factor (GF)^3^ domain of ∼110 residues (3–5). In the endoplasmic reticulum, two monomers noncovalently associate, and both the prodomain and GF domain are covalently linked into disulfide-linked dimers. At the same time, the TGF-β prodomain also associates with and becomes disulfide-linked to “milieu molecules,” such as latent TGF-β–binding protein and glycoprotein-A repetitions predominant protein (GARP) (6–9). After transport to the Golgi, the polypeptide connection between the prodomain and GF is cleaved by a proprotein convertase. However, the GF remains strongly noncovalently bound to the prodomain, which keeps it latent during storage in extracellular milieus. Prodomain–GF interfaces are important not only for latency but also for biosynthesis. The prodomain is required for proper folding and dimerization of the GF domain (10, 11). The C-terminal GF domain folds either concomitantly with or subsequently to the N-terminal prodomain (10, 12, 13).

The structure of the pro-TGF-β1 dimer reveals that the prodomain dimer surrounds the GF dimer (14). Two arm domain monomers with a jelly roll, β-sandwich fold dimerize and are disulfide-linked at a bowtie knot and surround the GF on one side. On the other side, the N and C termini of the prodomain form a straitjacket that more loosely surrounds the GF. Each straitjacket monomer contains an α1-helix that intercalates between the two GF monomers, a latency lasso that wraps around the distal ends of each GF monomer, and an α2-helix that snuggles against the GF–arm domain interface. TGF-β cannot bind its receptors and signal until it is released from the prodomain (*i.e.* activated) (15–17). TGF-β1 and -β3 are activated by integrins that bind to an RGDL*XX*(I/L) motif in the prodomain (18). The motif locates to a long loop in the arm domain called the bowtie tail, because it follows the two bowtie cysteines that disulfide-link the two prodomain monomers. A structure of an α_V_β_6_ integrin head bound to one monomer of a TGF-β dimer showed that integrin binding stabilizes an alternative conformation of the bowtie tail (19). Activation by integrin α_V_β_6_ requires force application by the actin cytoskeleton, which is resisted by the milieu molecule, resulting in distortion of the prodomain and release of the GF (9, 15, 16, 19).

The dimeric pro-TGF-β1 crystal structure suggested that there might be a swap between the prodomain and GF domain (14). In the structure, the straitjacket and arm domain on one side of the dimer dyad axis interacted much more with the monomer on the same side than with that on the other side of the dyad axis. The structure that suggested swapping was of mature pro-TGF-β1, which was cleaved between each prodomain and GF monomer. The C terminus of one arm domain was closer to the N terminus of one GF monomer than the other, and if these were linked in the original monomer, this monomer would correspond to the arm domain and GF on opposite sides of the dyad axis, with the smallest amount of noncovalent association in the final structure (14). In other words, there would have been a swap.

Here, we have determined a structure of pro-TGF-β1 with the PC cleavage site mutated, to mimic the structure of the TGF-β1 proprotein before PC cleavage. The previous structure of the α_V_β_6_ integrin head bound to one monomer of a TGF-β dimer also utilized PC cleavage site–mutated pro-TGF-β1 (19); however, there was no description of the structure around the cleavage site. Furthermore, the complex was less well resolved (3.5 Å) than the current pro-TGF-β1 structure (2.9 Å) and had more residues missing in density adjacent to the PC cleavage site. Our structure and our analysis of the complex structure support arm domain–GF domain swapping. Swapping also has important implications because it can provide a mechanism for preferential formation of heterodimers over homodimers when a cell synthesizes monomers for two different TGF-β family members. Such heterodimers can display unique biological activities, as in the case of BMP-2/7 heterodimers (20), and can alter activity, as in the case of inhibin heterodimers compared with activin homodimers (12).

To gain a more comprehensive understanding of pro-TGF-β1 structure, we also describe differences among three pro-TGF-β1 crystal structures that appear unrelated to prodomain cleavage and relate to differences in crystal lattice contacts and flexibility of specific regions in pro-TGF-β1. Analysis of structural changes in the α_V_β_6_ integrin complex with pro-TGF-β1 was limited to the region of contact between α_V_β_6_ integrin and pro-TGF-β1 in the 5-page report (19). Here, we take advantage of two previous structures containing pro-TGF-β1 and the current one to provide the first comprehensive analysis of structural differences. These structural differences are relevant to TGF-β activation because rigidity is important for force transmission, and flexibility is important for release of the GF from the prodomain and for the ability of pro-TGF-β1 to covalently and noncovalently associate with structurally distinct milieu molecules (6–9).

## Results

### Crystal structure of pro-TGF-β1 before furin cleavage

**Figure 1.**
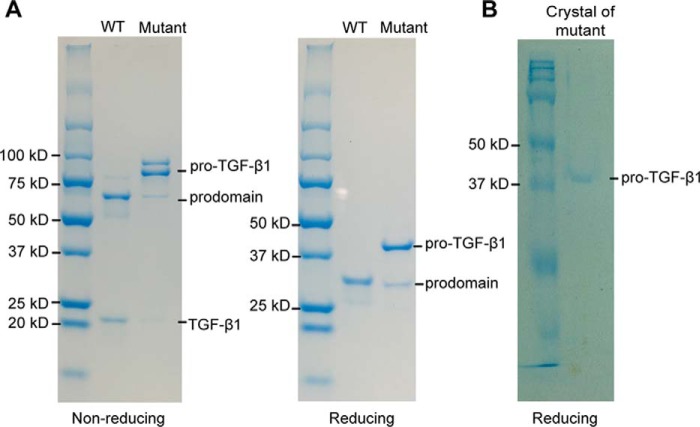
**SDS-PAGE of purified pro-TGF-β1 and crystal**. *A*, non-reducing (*left*) and reducing (*right*) SDS 4–20% PAGE of purified WT and R249A mutant pro-TGF-β1. *B*, reducing SDS-PAGE (10%) of a crystal of pro-TGF-β1 R249A mutant. Gels were stained with Coomassie Brilliant Blue.

We introduced an R249A mutation into the RHRR^249^ PC cleavage site between the prodomain and GF domain of human pro-TGF-β1. SDS-PAGE showed that the R249A mutant is >90% uncleaved, whereas WT protein is almost completely cleaved (Fig. 1*A*). Reducing SDS-PAGE of R249A mutant crystals showed a band corresponding to uncleaved pro-TGF-β1 monomer (Fig. 1*B*), whereas porcine pro-TGF-β1 crystals show predominantly the cleaved prodomain (14).

**Figure 2.**
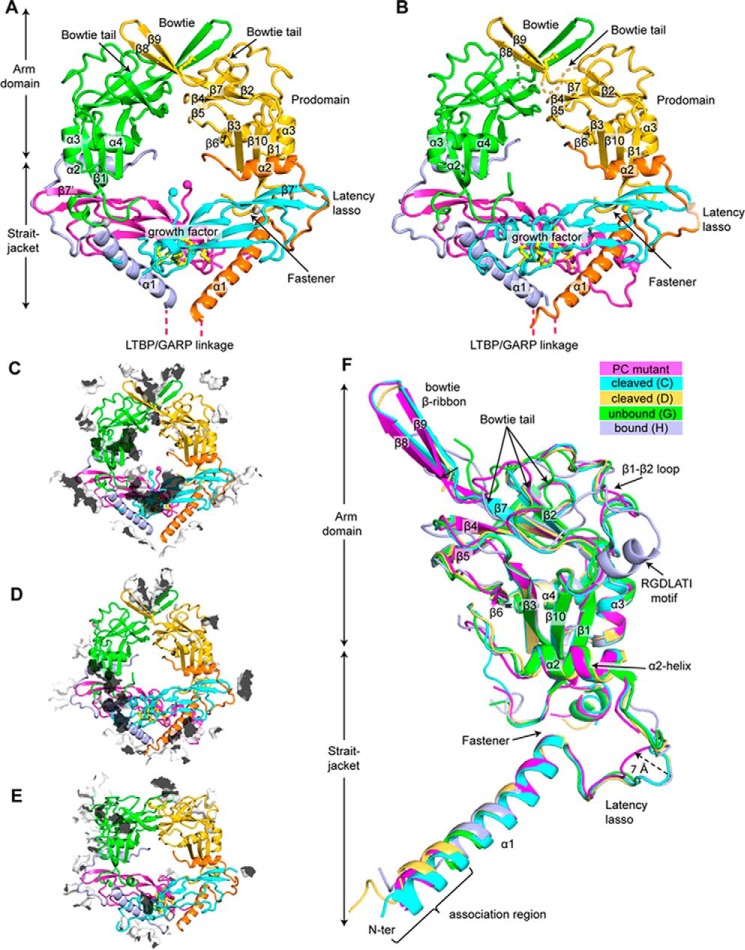
**Crystal structures, lattice contacts, and structural comparisons.** *A* and *B*, crystal structure of pro-TGF-β1 R249A mutant (*A*) and re-refined crystal structure of WT cleaved porcine pro-TGF-β1 (*B*). A *ribbon cartoon* is *colored differently* for straitjacket, arm, and GF domains. Disulfide bonds (*yellow*) are shown in *stick representations*. C termini of the prodomains and the N termini of the GFs are shown as *spheres. C–E*, lattice contacts in the crystal structures of uncleaved pro-TGF-β1 R249A PC mutant (*C*), cleaved pro-TGF-β1 WT (*D*), and uncleaved pro-TGF-β1 R249A PC mutant in complex with integrin α_V_β_6_ (*E*). Crystal lattice contacts are shown as *transparent surfaces* with their *outsides white* and *insides black. F*, comparison of five representative pro-TGF-β1 prodomain monomers: the human R249A mutant 2.9 Å structure (PC mutant), WT porcine (cleaved chains C and D), and integrin-bound and unbound human R249A mutant monomers in a pro-TGF-β1 complex structure (19) (unbound chain H and bound chain G).

We determined a crystal structure to a resolution of 2.9 Å of human R249A mutant pro-TGF-β1 (Table 1 and Fig. 2*A*). The structure contains one monomer in the asymmetric unit; the other monomer of the dimer shown in the figures is a symmetry mate. We also reprocessed to higher resolution and re-refined the previously described, cleaved porcine pro-TGF-β1 structure (Table 2 and Fig. 2*B*). Despite the increase in resolution from 3.05 to 2.9 Å, the overall quality of the re-refined model and its *R*_free_ greatly improved (Table 2). The porcine, PC-cleaved structure has four crystallographically distinct monomers in the asymmetric unit but nonetheless has pseudosymmetry that resembles the symmetry of the human R249A mutant. Despite these overall similarities, the crystal lattice contacts show marked differences (Fig. 2, *C* and *D*).

**Table 1:** **Statistics of X-ray diffraction and structure refinement of human pro-TGF-β1 R249A mutant (PDB entry 5VQP)**

	Pro-TGF-β1 R249A mutant (PDB entry 5VQP)
**Data collection statistics**	
Space group	P 6 2 2
α, β, γ (degrees)	90, 90, 120
Unit cell (*a*, *b*, *c*), Å	104.4, 104.4, 141.9
Resolution range (Å)	50.0–2.9 (3.0–2.9)*^a^*
Completeness (%)	99.7 (99.9)*^a^*
No. of unique reflections	10,672 (1,024)*^a^*
Redundancy	10.5 (10.9)*^a^*
*R*_merge_*^b^* (%)	17.1 (601)*^a^*
*I*/σ	12.3 (0.5)*^a^*
*CC*½ (%)*^c^*	99.9 (15.1)*^a^*
Wavelength (Å)	1.0332

**Refinement statistics**	
*R*_work_*^d^* (%)	25.2 (41.3)*^a^*
*R*_free_*^e^* (%)	29.3 (42.3)*^a^*
Bond RMSD (Å)	0.004
Angle RMSD (degrees)	0.64
Ramachandran plot*^f^* (favored/allowed/outlier)	95.5/4.5/0
MolProbity percentile*^f^* (Clashscore/geometry)	96/99
No. of atoms	
Protein	2576
Carbohydrates	39
No. of *cis*-prolines	1
*B*-factors	
Protein	127.0
Carbohydrates	221.8

*^a^* Numbers in parentheses are for the highest-resolution shell.

*^b^ R*_merge_ = Σ*_h_* Σ*_i_*|*Ii*(*h*) − 〈*I*(*h*)〉|/Σ*_h_*Σ*_i_ Ii*(*h*), where *Ii*(*h*) and 〈*I*(*h*)〉 are the *i*th and mean measurement of the intensity of reflection *h*.

*^c^* Pearson's correlation coefficient between average intensities of random half-data sets for unique reflections (34).

*^d^ R*_work_ = Σ*_h_*‖*F*_obs_(*h*)| − |*F*_calc_(*h*)‖/Σ*_h_*|*F*_obs_(*h*)|, where *F*_obs_(h) and *F*_calc_(*h*) are the observed and calculated structure factors, respectively. No *I*/σ(*I*) cutoff was applied.

*^e^ R*_free_ is the *R* value obtained for a test set of reflections consisting of a randomly selected ∼3% subset of the data set excluded from refinement.

*^f^* Calculated with MolProbity (22).

**Table 2:** **Statistics of X-ray diffraction and re-refinement of porcine pro-TGF-β1**

	Re-refined pro-TGF-β1 WT (PDB entry 5VQF)	Original pro-TGF-β1 WT (PDB entry 3RJR)
**Data collection statistics**		
Space group	P 2_1_	P 2_1_
α, β, γ (degrees)	90, 96.7, 90	90, 96.7, 90
Unit cell (*a*, *b*, *c*), Å	54.7, 126.9, 137.9	54.7, 127.1, 138.2
Resolution range (Å)	50.0–2.9 (3.0–2.9)*^a^*	50.0–3.05 (3.21–3.05)*^a^*
Completeness (%)	96.1 (97.8)*^a^*	97.9 (96.5)*^a^*
No. of unique reflections	39,918 (4,031)*^a^*	35,401 (4857)*^a^*
Redundancy	4.3 (4.3)*^a^*	5.6 (4.2)*^a^*
*R*_merge_*^b^* (%)	5.2 (274)*^a^*	4.7 (75.2)*^a^*
*I*/σ	15.3 (0.6)*^a^*	16.4 (1.8)*^a^*
*CC*½ (%)*^c^*	99.9 (32.6)*^a^*	NA*^d^*
Wavelength (Å)	1.0332	1.0332

**Refinement statistics**		
*R*_work_*^e^* (%)	23.9 (45.2)*^a^*	27.4 (36.7)*^a^*
*R*_free_*^f^* (%)	28.0 (52.0)*^a^*	31.1 (41.9)*^a^*
Bond RMSD (Å)	0.005	0.002
Angle RMSD (degrees)	0.85	0.488
Ramachandran plot*^g^* (favored/allowed/outlier)	96.7/3.3/0	88.2/11/0.8
MolProbity percentile*^g^* (Clashscore/geometry)	98/100	97/98
No. of atoms		
Protein	10,658	10,843
Carbohydrates	95	112
No. of *cis*-prolines	4	4
*B*-factors		
Protein	188.4	214.4
Carbohydrates	266.4	257.4

*^a^* Numbers in parentheses are for the highest resolution shell.

*^b^ R*_merge_ = Σ*_h_*Σ*_i_*|*Ii*(*h*) − 〈*I*(*h*)〉|/Σ*_h_*Σ*_i_Ii*(*h*), where *Ii*(*h*) and 〈*I*(*h*)〉 are the *i*th and mean measurement of the intensity of reflection *h*.

*^c^* Pearson's correlation coefficient between average intensities of random half-data sets for unique reflections (34).

*^d^* NA, not applicable.

*^e^ R*_work_ = Σ*_h_*‖*F*_obs_(*h*)| − |*F*_calc_(*h*)‖/Σ*_h_*|*F*_obs_(h)|, where *F*_obs_(*h*) and *F*_calc_(*h*) are the observed and calculated structure factors, respectively. No *I*/σ(*I*) cutoff was applied.

*^f^ R*_free_ is the *R* value obtained for a test set of reflections consisting of a randomly selected ∼3% subset of the data set excluded from refinement.

*^g^* Calculated with MolProbity (22).

### Structurally conserved and variant regions of pro-TGF-β1

We first describe the overall structure of furin-mutant pro-TGF-β1 and compare it with previous pro-TGF-β1 structures before focusing on structural alterations around the PC cleavage site. Comparisons give insights into regions of pro-TGF-β1 that are flexible and hence may be affected by lattice contacts in crystals, as opposed to regions of pro-TGF-β1 that are more structurally constrained. Studying these structural features is important in understanding pro-TGF-β1 biology, including reshaping of the straitjacket region by applied force to release the GF in TGF-β1 activation, force transmission from the integrin binding site through the arm domain to the straitjacket, and the ability of the N-terminal portion of the prodomain to noncovalently associate with and disulfide-link to structurally distinct milieu molecules (6–9). A single crystallographically unique furin-mutant pro-TGF-β1 is present in the current structure. Four unique monomers are present in re-refined, cleaved, porcine pro-TGF-β1; because chains A and C and B and D are similar to one another, we only show chains C and D in the figures. Four unique monomers are present in the α_V_β_6_ complex with furin-mutant pro-TGF-β (19); because integrin-bound monomers D and H are similar and unbound monomers C and G are similar, we only show chains G and H in the figures. These five prodomain monomers are overlaid in Fig. 2*F*.

In the prodomain arm domain, β-strands β1 to β7 of the jelly roll β-sandwich fold are structurally invariant, as are major α-helices α2–α4 of the arm domain, as shown in the overlay of the five representative prodomain monomers (Fig. 2*F*). β-Sheet domains are relatively resistant to force. The relatively rigid arm domain β-sandwich fold thus enables integrins bound to the RGDLATI motif to transmit force through the arm domain to the straitjacket elements that surround the TGF-β1 GF (19).

Large structural alterations upon integrin binding in the remarkably long 30-residue bowtie tail that contains the integrin-binding RGDLATI motif have been described previously (19). In the human PC-mutant monomer described here, all residues in the bowtie tail can be traced, whereas 6–9 residues are disordered in previous structures. However, density is poor in some regions of the PC-mutant bowtie tail, consistent with large variation in this region between structures. The two-stranded bowtie β-ribbon in porcine PC-cleaved and human uncleaved pro-TGF-β1 (Fig. 2, *A* and *B*) is a consequence of formation of a four-stranded super-β-sheet with a two-stranded bowtie β-ribbon from a neighboring molecule related by symmetry or pseudosymmetry. In the absence of such a lattice contact, no β-ribbon is present, and nine residues are disordered in the integrin-unbound monomer from the α_V_β_6_ complex (19). Notably, the β1-β2 loop that neighbors the bowtie tail is also highly structurally variable (Fig. 2*F*). This loop corresponds to a long meander between arm domain β-strands 1 and 2. Backbone movement in the meander should facilitate reshaping of the neighboring bowtie tail.

In the straitjacket, the C-terminal portion of the prodomain α1-helix, which inserts between the two GF domain monomers, is highly conserved in position. The C-terminal end of the α1-helix interacts with residues 74–76 to form a fastener that surrounds one GF monomer; each portion of the fastener is also highly conserved in position (Fig. 2*F*). The α2-helix, which interacts with both the GF and the arm domain, is also highly conserved structurally among the five representative pro-TGF-β1 monomers. This conservation in position of the two fastener elements and the α2-helix correlates with the observation that they are the most force-resistant straitjacket elements when integrin and actin cytoskeleton–dependent pulling on pro-TGF-β1 is resisted by the α1-helix residues that link to milieu molecules (*i.e.* fastener and α2-helix disruption correlates with peaks in force during pulling) (19).

In contrast, the latency lasso and N terminus of the prodomain are highly variable among representative straitjacket structures. In the human R249A mutant pro-TGF-β1 structure, the latency lasso is displaced up to 7 Å by a lattice contact relative to other structures (*arrow* in Fig. 2*F*), and two residues are disordered. Almost the entire latency lasso, residues 33–45, differs by 2 Å or more among the structures, showing that the lasso only loosely wraps around the GF. At the N terminus, residues 1–2 are disordered in the human R249A mutant. The following α1-helix is stabilized as α-helical by contacts with the α1-helix of a symmetry-related molecule in the crystal lattice, as also occurred in the previous porcine, PC-cleaved pro-TGF-β1 structure (14). In the absence of such lattice contacts in the integrin complex with pro-TGF-β1 (19), residues 1–9 are disordered or differ in conformation. Prodomain residues 1–9 have no contact with other portions of pro-TGF-β1 and correspond to an “association region” that contains Cys-4 that disulfide-links to latent TGF-β–binding protein or GARP. Because these milieu molecules have no structural similarity, the association region must be able to adopt different conformations to noncovalently associate with them. Furthermore, one pro-TGF-β1 dimer associates with a single milieu molecule in asymmetric 2:1 complexes. Thus, the association regions of the two monomers bound to a milieu molecule must adopt different conformations to bind to distinct portions of a milieu molecule.

### The structure around the PC cleavage site

**Figure 3.**
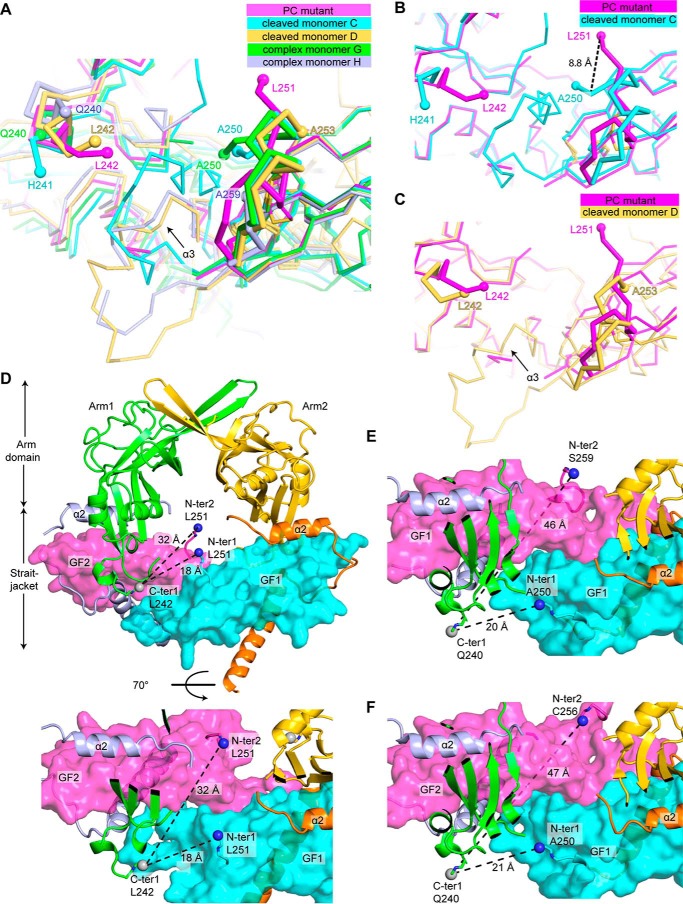
**Prodomain–GF connections.** *A–C*, overlays of the PC cleavage region in different crystal structures: R249A PC mutant 2.9 Å structure chain A (PC mutant), WT porcine cleaved chains C and D (cleaved monomer C and cleaved monomer D), and integrin complex R249A PC mutant chains G and H (complex monomer G and complex monomer H). *A*, all five structures; *B* and *C*, individual comparisons of uncleaved and cleaved monomers. *D*, views of the two possible arm domain–GF connections in the TGF-β1 R249A PC mutant 2.9 Å structure, from terminal residues in one arm domain monomer (C-ter 1) to terminal residues in one GF monomer (N-ter 1) or the other monomer (N-ter 2). The gaps to be spanned are *dashed. Spheres* mark the carbon atom of C-ter 1 and nitrogen atoms of N-ter 1 and N-ter 2. Terminal residues are *labeled*. Prodomain and GF domains are shown in *ribbon cartoon* and *surface representations*, respectively. *E* and *F*, two possible arm domain–GF connections in integrin complex uncleaved pro-TGF-β1 R249A dimers. *E*, chains G+H; *F*, chains C+D. Views are identical to those in the *bottom view* in *D*, and other details are as in *D*.

The conformation of the growth factor domain N terminus in the R249A mutant pro-TGF-β1 monomer differs markedly from the two representative monomers in cleaved pro-TGF-β1 (Figs. 2 (*A* and *B*) and 3 (*A–C*)). In the structures reported here, the terminal residues in the prodomain and GF bordering the PC cleavage region have main-chain density in simulated annealing composite omit maps contoured at 1 σ (Fig. S1). In the uncleaved R249A mutant 2.9 Å structure, we can trace the electron density for the polypeptide chain up to prodomain residue 242. After seven prodomain residues and the N-terminal GF residue missing in density, the trace of the GF begins with its second residue, residue 251. Residue 251 is present in the solvent-filled cavity between the arm domains and the GF in the center of the ring-shaped pro-TGF-β1 dimer (Figs. 2*A* and 3 (*A–D*)). Residues 251–253 lack any stabilizing van der Waals or hydrogen bond interactions with neighboring residues. A neighboring molecule in the crystal lattice comes close to residue 253 (Fig. 2*C*) and decreases the space accessible to residues 251–253 but makes no specific contacts. The ordering of residues 251 and 252 in the absence of any interactions with visualized residues suggests that their position is stabilized by the linkage of residue 251 to disordered residues 243–250, which connect to ordered prodomain residue 242.

In the mature, cleaved GF, the N terminus has a distinct conformation (Figs. 2*B* and 3 (*A–C*)). Residues 252–258 in the mature GF are α-helical, in contrast to the much more extended conformation in the uncleaved GF. The conformations differ among the N termini of the crystallographically distinct GF monomers in cleaved WT pro-TGF-β1. Chains A and C show ordering beginning with N-terminal GF residue 250 (Fig. 3*B*). Chains B and D show ordering beginning with GF residue 253 (Fig. 3*C*). Nonetheless, the structural environments of the N-terminal residues of the cleaved GFs are similar overall in having close interactions with other GF residues, and none extend into the solvent-filled channel in the middle of the pro-TGF-β1 ring. The N terminus of the mature GF extends toward the α3-helix of the GF, which is highly variable in position in cleaved TGF-β1 pro-complexes and is disordered in the uncleaved GF of the R249A mutant pro-TGF-β1. In concert with movement of the α3-helix, the N terminus of the mature GF moves so that it remains in van der Waals contact with the GF α3-helix. As a consequence of the extension of residues 251–254 in uncleaved pro-TGF-β1 toward the solvent channel in the ring instead of toward the α3-helix, residue 251 adopts positions that differ by 8.8 Å in uncleaved compared with cleaved pro-TGF-β1 (Fig. 3*B*).

The overall structures of WT and R249A mutant pro-TGF-β1 show that there are no large-scale conformational changes in the prodomain or GF or their orientation with respect to one another that relate to separation of the prodomain from the GF by PC cleavage. The most significant difference localizes to the N terminus of the GF domain, which, as described above, is altered in orientation in the absence of cleavage. Eight residues (positions 243–250) linking the prodomain to the growth factor domain are missing in density, consistent with the need of flexibility to fit into the catalytic site of the PC protease.

We further examined the previously undescribed structure around the PC cleavage site in the two representative monomers (chains G and H) of pro-TGF-β1 complexes with integrin α_V_β_6_ (Fig. 3*A*). Superposition of chains G and H from the complex on chain A from R249A mutant pro-TGF-β1 crystallized alone reveals further flexibility. In chains G and H, residues 241–258 and 241–249, respectively, are disordered, compared with residues 243–250 in chain A of pro-TGF-β1 alone (Fig. 3*A*). However, the positions of ordered residues 240 and 251–261 also differ markedly (Fig. 3*A*), correlating with the different lattice environments of the three R249A mutant monomers compared (Fig. 2, *C* and *E*). Among the three monomers, differences of up to 2 Å in Cα position begin at prodomain residue Gln-240 and end at GF residue Glu-261. Thus, residues 240–261 (22 residues) are capable of remodeling to extend away from pro-TGF-β1 and fit into the PC cleavage site.

### Evidence for arm domain and growth factor swapping

**Figure 4.**
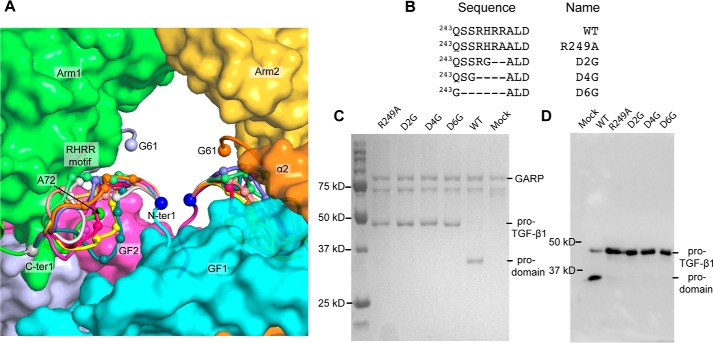
**Modeling and mutagenesis of the arm domain–GF linker region.** *A*, representative arm domain–GF connections (residues 243–250) built by MODELLER between the C-ter 1–N-ter 1 connection are shown as loops of different *colors* with Cα atoms of the RHRR PC cleavage site shown with *small spheres*. Residues 61 and 72, adjacent to the residues missing in density between the straitjacket and arm domain, are also marked with *spheres* (see “Discussion”). The remaining portions of pro-TGF-β1 are shown as *surface representations. B*, PC site mutations. *C*, WT and mutant pro-TGF-β1 co-expression with soluble GARP. Culture supernatants were incubated with StrepTactin-Sepharose (GE Healthcare) for 1 h. StrepTactin beads were washed and heated at 100 °C in reducing SDS sample buffer, and eluates were subjected to SDS-PAGE and stained with Coomassie Brilliant Blue. *D*, transient expression of WT and mutant pro-TGF-β1. Culture supernatants were subjected to reducing SDS-PAGE and Western blotting with antibody to the TGF-β1 prodomain (BAF246, R&D Systems).

We used modeling and mutagenesis to establish which prodomain–GF connection in the 2.9 Å R249A mutant structure was physiologic. The most C-terminal residue with electron density in one prodomain (labeled *C-ter 1* in Fig. 3*D*) must link through disordered residues either to the N terminus of one GF monomer (N-ter 1; a distance of 18 Å) or to the N terminus of the other monomer (N-ter 2; a distance of 32 Å). Whereas a direct connection between C-ter 1 and N-ter 1 can be built over the 18-Å distance, the connection to N-ter 2 of 32 Å cannot be built straight, because fastener residues 72–74 and the GF dimer are in the way, and the polypeptide must curve around them as it goes through the central cavity of pro-TGF-β to connect. To test the feasibility of the alternative arm domain–GF connections, we built the residues missing in density using Modeler (21). As reported by MolProbity (22), the 32-Å distance could not be spanned without introducing substantial clashes, bad geometry, and bond length outliers (*i.e.* chain breaks). The 32-Å connection showed 100% bond length outliers over residues 242–251 for 20 of 20 models. As a further test, validation for deposition to the Protein Data Bank reported that the 32-Å connection was too long to be spanned for the number of missing residues. Moreover, lack of flexibility in the 32-Å connection would prohibit the remodeling required to fit into the PC protease active site cleft and would position the cleavage site in the center of the cavity of the pro-TGF-β ring, where it would be inaccessible to the large 90-kDa PC protease. In contrast, the 18-Å distance could easily be spanned by multiple conformations of the prodomain–GF linker, including those with the RHRR^249^ PC recognition motif well exposed to solvent (Fig. 4*A*). Representatives of the 20 connections built by Modeler between C-ter 1 and N-ter 1 are shown in *different colors* in Fig. 4*A*, with the four residues in the PC recognition motif shown as *small C*α-*atom spheres*.

The positions of C-ter 1, N-ter 1, and N-ter 2 and their relation to the structural environment are shown for the pro-TGF-β1 integrin complex structure in Fig. 3, *E* and *F*. Additionally, the terminal residues visualized in density and the distance that would be needed to connect the terminal residues (assuming a straight extended chain could be built) are compared in Table 3. The results show that the C-ter 1 and N-ter 1 linkages (between prodomain and GFs with the same chain IDs) are feasible, given the distances to be spanned and the number of missing residues (Table 3). In contrast, the distances to be spanned are always longer for C-ter 1 to N-ter 2 linkages (between prodomain and GFs with different chain IDs in Table 3). The number of missing residues is insufficient to span the required distance between C-ter 1 and N-ter 2; the straight line measurements in Table 3 are underestimates, because the straight lines go through the GF domains, as shown in Fig. 3 (*E* and *F*). Overall, the model building for the R249A pro-TGF-β1 structure and structural analysis of the complex structure demonstrate that only one arm domain–GF connection, C-ter 1 to N-ter 1, is possible. Furthermore, comparisons among the structures in different crystal lattice environments demonstrate that flexibility around the cleavage site extends beyond residues that are disordered in the R249A pro-TGF-β1 structure and includes residues 240–261.

**Table 3:** **Terminal residues bordering the PC cleavage site connectivity region and distances in the dimer structures**

Structure	Prodomain C terminus	Growth factor N terminus	Terminal residues in each chain	Missing residues	Distance
					Å
Pro-TGF-β1 R249A mutant (5VQP)	Chain A, Leu-242	Chain A, Leu-251	A, Leu-242; A, Leu-251	8	18
			A, Leu-242; Sym*^a^*/Leu-251	8	32
Pro-TGF-β1 dimer (G+H) in complex with integrin (5FFO)	Chain G, Gln-240	Chain G, Ala-250	G, Gln-240; G, Ala-250	9	20
			G, Gln-240; H, Ser-259	18	46
	Chain H, Gln-240	Chain H, Ser-259	H, Gln-240; H, Ser-259	18	23
			H, Gln-240; G, Ala-250	9	39
Pro-TGF-β1 dimer (C+D) in complex with integrin (5FFO)	Chain C, Gln-240	Chain C, Ala-250	C, Gln-240; C, Ala-250	9	21
			C, Gln-240; D, Cys-256	15	47
	Chain D, Gln-240	Chain D, Cys-256	D, Gln-240; D, Cys-256	15	20
			D, Gln-240; C, Ala-250	9	39

*^a^* Symmetry-related molecule.

To further characterize the prodomain–GF connection, we shortened it by deleting 2–6 residues. We hypothesized that the physiologic connection should be flexible to enable PC cleavage. Thus, we expected that the N-ter 1–C-ter 1 connection should be capable of being shortened, without disrupting proper folding of pro-TGF-β1. Because mammalian cells have endoplasmic reticulum quality control systems that require proper protein folding before secretion, we assayed for the effect of deletion mutations on pro-TGF-β1 secretion by 293T cell transfectants. We deleted 2, 4, or 6 residues, including the RHRR cleavage site, and introduced a glycine substitution to enable flexibility at the deletion position in the D2, D4, and D6 mutations (Fig. 4*B*). These mutations abolished PC cleavage and yet had no effect on the level of pro-TGF-β1 expression (Fig. 4, *C* and *D*). The ability to substantially shorten the linker without affecting expression (*i.e.* folding) supports the shorter of the two connections.

## Discussion

We describe here the crystal structure of a pro-TGF-β1 R249A mutant that is uncleaved by PC and contrast it with a re-refined structure of cleaved pro-TGF-β1. Clear differences are present adjacent to the cleavage site in at least three N-terminal GF residues. PC cleavage appears to result in no overall conformational changes in procomplex structure, although interesting differences are present distal from the cleavage site that appear to be influenced by lattice contacts that differ among structures. We also examine the previously undescribed structural features of an R249A mutant that was included in a complex structure with the head of integrin α_V_β_6_ (19). The pro-TGF-β1 R249A mutant structure determined here is at higher resolution and has fewer residues missing in density in the PC cleavage region and thus provides a more definitive analysis of swapping; however, the complex structure confirms and extends the conclusions. The R249A mutant is a model for the structure of the pro-form of pro-TGF-β1 present during biosynthesis, after folding is completed in the endoplasmic reticulum, and before cleavage by a PC protease in the Golgi. As such, it provides the closest glimpse yet available for the connectivities between prodomains and GF domains within individual pro-TGF-β1 monomers during biosynthesis.

**Figure 5.**
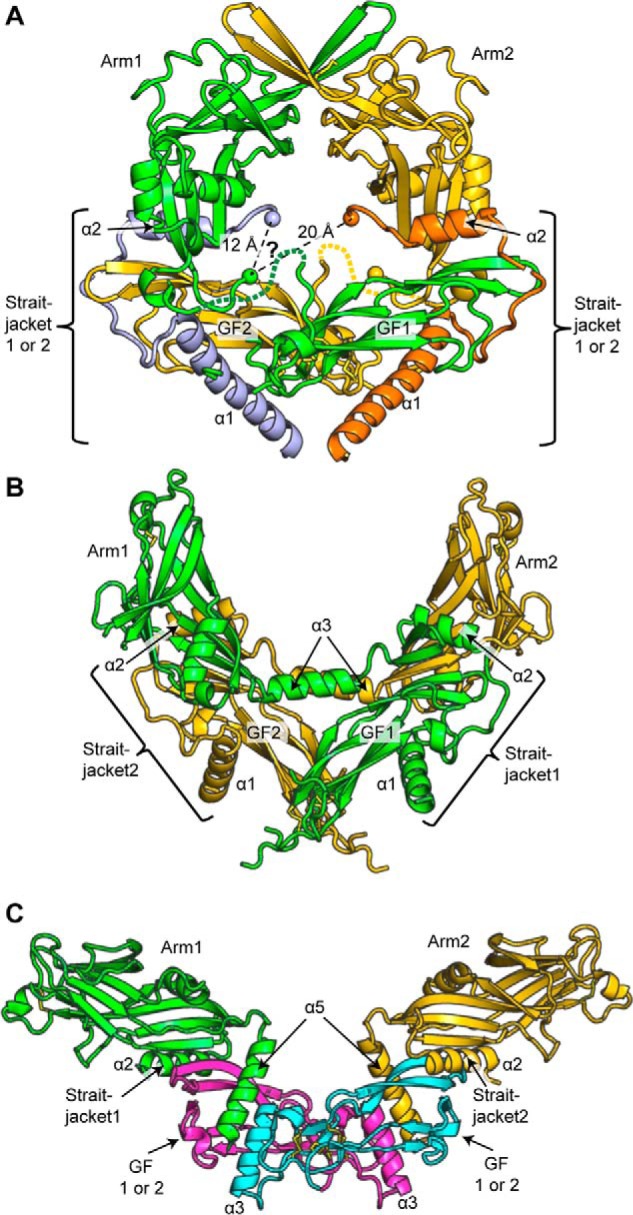
**Prodomain–GF swapping.** *A–C*, straitjacket, arm domain, and GF connectivity in pro-TGF-β1 (*A*), pro-activin A (*B*), and pro-BMP9 (*C*). Straitjacket, arm, and GF elements are shown in the *same color* if they belong to the same precursor monomer; elements are numbered *1* or *2* according to their monomers. They are labeled *1* or *2* when the monomer from which they are derived is unknown and are given a *distinctive color* (straitjackets in pro-TGF-β1 and GFs in pro-BMP9).

SDS-PAGE of purified pro-TGF-β1 R249A mutant protein and crystals formed from it, along with the distinct conformation of the GF N terminus, show that the prodomain–GF linker region was intact in this structure, although the electron density was too weak to be traced. Distance measurements and modeling based on this structure unambiguously define the shortest of two possible prodomain–GF connections as that which is physiologic. These results were further supported by the demonstration that shortening of the prodomain–GF linker by deletion of 2–6 residues had no adverse impact on the amount of pro-TGF-β1 biosynthesis or secretion. We therefore concluded that the prodomain arm domain is connected to the GF with which it has no noncovalent interaction. Arm domains and GF domains that are derived from the same precursor monomers are shown in the *same color* in Fig. 5*A*. The arm domain noncovalently interacts with the GF domain derived from the other precursor monomer and forms a super-β-sheet with it; the total binding interface is 375 Å^2^.

Our results also explain why arm-GF swapping is found and intramonomer association does not occur. The arm and GF within a precursor monomer could not associate with one another to form a super-β-sheet as described in the previous paragraph for the arm and GF domains from different monomers, because, as we have shown, the distance, which corresponds to the N1-C2 connection in the dimer, is too long to be spanned. Many other factors that favor swapping may also come into play, including maximizing the entropy of the polar residues around the cleavage site by not straightening them and not confining them to the narrow channel in the middle of the pro-TGF-β1 ring. The channel is more crowded than it appears in our figures, because it also includes disordered residues that connect Gly-61 in the straitjacket to Ala-72 in the arm domain (Fig. 4*A*). Arm domain–GF domain swapping must occur in the endoplasmic reticulum concomitant with protein folding and formation of intra- and intermonomer disulfide bonds and before PC cleavage in the Golgi.

As there was little discussion in a paper on an integrin complex on structural variation in pro-TGF-β1 aside from that associated with integrin binding (19), we have described this variation here and extended it to the newly determined pro-complex structure. The latency lasso and association region are remarkably malleable. In contrast, other portions of the straitjacket, including the C-terminal portion of the α1-helix, its interaction with the fastener, and the α2-helix, are structurally conserved. The arm domain jelly roll fold is also structurally conserved. These observations correlate with the dynamics of these regions measured by hydrogen–deuterium exchange (19). We have further observed that residues 240–261 flanking the PC cleavage site at Arg-249 are either disordered or variable in structure in different lattice environments. The sequence in this region, ^240^QSSRHR(R/A)^249^ALDTNYCSSTE^261^ is largely polar and includes a Cys residue in a partially disordered disulfide bond.

It is interesting to examine our conclusions on prodomain–GF connectivity in pro-TGF-β1 in light of recent structures of pro-complexes for BMP-9 (23) and activin (24). In pro-TGF-β1, we have shown that the arm domain and the GF domain more distal in the dimeric structure are derived from the same monomer. The arm and GF domains derived from one monomer are *colored green*, and those from the other monomer are in *gold* in Fig. 5*A*. Whereas the connection between the arm domain and the GF in precursor monomers has been assigned here, the connection between the straitjacket and the arm domain is undefined. In pro-TGF-β1, the straitjacket shows density from the α1-helix through the latency lasso through to the end of the α2-helix. There is then a gap, with density missing between residues 61 and 72 that connect the straitjacket to the arm domain. The disordered residues lie in the central solvent-filled cavity in the pro-TGF-β1 ring (Figs. 2*A*, 4*A*, and 5*A*). Whether the straitjacket connects to the arm domain on the other side of the dimer or on the same side is not known. In the uncleaved R249A structure, the *light blue-colored* straitjacket would connect to the *green* arm domain over a distance of 12.2 Å, and the *orange* straitjacket on the other side of the dimer would connect to the *green* arm domain over a distance of 20.2 Å (*C*α *spheres* and *dashed lines* in Fig. 5*A*). Either distance can be spanned by the 10 missing residues. Therefore, it is not possible to know whether the straitjacket and arm domain that are close or distal to one another are in the same polypeptide chain, and chain IDs for the straitjacket region in current pro-TGF-β1 structures are arbitrary.

For pro-activin A, structures were determined with and without cleavage at a 3C protease site that replaced the PC site (24). The prodomain–GF linker was disordered in both structures; however, based on the distance to be spanned, it was concluded that the arm domain–GF connection was the same as described here for pro-TGF-β1 (Fig. 5*B*). In other words, in both pro-TGF-β1 and pro-activin A, the arm domain connects to the GF with which it has no noncovalent interaction and intimately noncovalently interacts with the GF in the other monomer. Interestingly, in pro-activin A, a highly ordered helix (α3) connects the straitjacket on one side of the dimer dyad axis to the arm domain on the opposite side (Fig. 5*B*). Thus, in Fig. 5*B*, each pro-activin A precursor monomer has a *single color* to show monomer connectivity. It can be seen that there are two types of swaps. In each monomer, the straitjacket connects to the arm domain on the opposite side of the dimer dyad axis; furthermore, each arm domain connects to the GF on the opposite side of the dyad axis. This type of swapping is also possible in pro-TGF-β1.

Pro-BMP-9 (23) is yet another case. It lacks density for the α1-helix and latency lasso yet shows good density for the α2-helix. Furthermore, the density between the α2-helix and the arm domain is continuous and connects the α2-helix to the same arm domain with which it noncovalently associates (shown in the *same color* in Fig. 5*C*). Thus, pro-BMP-9 lacks the swap between the straitjacket and the arm domain seen in pro-activin A. However, the α1-helix and latency lasso are missing in density in pro-BMP9, and therefore it is important to consider whether a putative straitjacket–arm domain swap present in the pro-form could have been reversed by conformational change after PC cleavage. The putative swap is in the loop between the straitjacket α2-helix and arm domain β1-strand. This loop is 12 residues longer in pro-TGF-β1 and 17 residues longer in pro-activin A than in pro-BMP-9 (2). Even building a model of pro-BMP-9 with a conformation in which the two arm domains associate closely, as in pro-TGF-β1 (23), it appears that the loop between the α2-helix and β1-strand is not long enough for such swapping to occur in pro-BMP-9. Thus, in pro-BMP-9, swapping between the straitjacket and arm domains is neither observed experimentally nor feasible in an alternative pro-complex conformation. It thus appears that the TGF-β family members activin A and BMP-9 differ in their straitjacket–arm domain swaps.

Swapping between the straitjacket, arm domain, and GF domain has profound biological implications for the TGF-β family at large. TGF-β family members form homodimers as well as heterodimers. Inhibin-β subunits homodimerize to form activins, such as pro-activin A, as discussed above, and heterodimerize with inhibin-α subunits to form inhibins (12, 25, 26). Some BMP heterodimers show higher activity than homodimers *in vitro* and *in vivo*, including BMP-4/7 (27–29) and BMP-2/7 (20, 30–32). Although structures of biologically relevant heterodimers are not available, the structure here of uncleaved pro-TGF-β1 together with structures of pro-activin A and pro-BMP-9 suggest that swapping of two elements of the prodomain with the GF domain could provide mechanisms for preferential formation of heterodimers over homodimers for some TGF-β family members. More specifically, we show that the arm domain of one monomer interacts with the GF domain of the other monomer. Thus, the arm domain of TGF-β family member 1 may have higher affinity for the GF in a second monomer of TGF-β family member 2 than a second monomer of identical family member 1. Similarly, the arm domain of family member 2 may have higher affinity for the GF in a second monomer of TGF-β family member 1 than in a second monomer of identical family member 2. In this scenario, when a cell co-expresses family members 1 and 2, during biosynthesis in the endoplasmic reticulum, formation of heterodimers between family members 1 and 2 will be favored over formation of homodimers of family member 1 or 2.

The TGF-β family is very diverse, with 33 genes giving rise to a larger number of homo- and heterodimers that regulate all aspects of development and homeostasis. The TGF-β superfamily is larger than other extracellular protein families that regulate development, including the Wnt, Frizzled, Delta/Jagged, and Notch families (2). Multiple families that contain similar cystine-knot fold GF domains also emerged at the dawn of metazoans, but the TGF-β family multiplied more than any other. TGF-β has a larger prodomain than any other cystine-knot fold cytokine, and it has been argued that this size, combined with the capacity for diversification of the prodomain, contributed to the evolutionary success of the TGF-β family (2). It has become increasingly clear that prodomains are of key importance in the physiology of the TGF-β family. The study here and comparisons with other pro-complexes extend our understanding of how TGF-β family prodomains interact with and regulate their GFs.

## Experimental procedures

### Protein expression and purification

The human pro-TGF-β1 expression construct contains an N-terminal His_8_ tag, followed by a streptavidin-binding protein tag and a 3C protease site (14). A C4S mutation and *N*-glycosylation site mutations N107Q and N147Q were introduced to facilitate protein expression, secretion, and crystallization. In addition, R249A, D2G, D4G, and D6G PC site mutations (Fig. 4*B*) were introduced. R249A mutant protein expression and purification were as described (19). Human pro-TGF-β1 constructs with no N-terminal tags, WT Cys-4 and R249A, D2G, D4G, and D6G PC site mutations (Fig. 4*B*), were also used for co-expression with GARP.

### Cell culture and transfection

HEK293S GnTI^−^ stable cell lines expressing soluble GARP protein with N-terminal His_8_ tag, streptavidin-binding protein tag, and a 3C protease site and HEK293T cells were maintained in DMEM supplemented with 10% FBS, 4 mm l-glutamine, and 1% non-essential amino acids. Cells were transiently transfected with WT and mutant pro-TGF-β1 constructs using Lipofectamine 2000 (Invitrogen) according to the manufacturer's instructions. Culture supernatants were harvested after 48 h to test for pro-TGF-β1 expression and co-expression with GARP.

### Crystal structures

Crystals of pro-TGF-β1 R249A mutant (1 μl, 10 mg/ml, 20 mm Tris-HCl, pH 8.0, 150 mm NaCl) were formed in hanging drops at 16 °C with 1 μl of 10% dioxane, 100 mm MES, pH 6.5, 1.7 m (NH_4_)_2_SO_4_ (well solution). Crystals were cryoprotected with well solution containing 31.4% Li_2_SO_4_. Diffraction data from GM/CA-CAT beamline 23-ID of the Advanced Photon Source at the Argonne National Laboratory were processed with XDS (33) with cross-correlation to determine the diffraction limit (34). Structures were solved with molecular replacement by PHASER (35) with cleaved pro-TGF-β1 (Protein Data Bank entry 3RJR) as the search model. The previous cleaved pro-TGF-β1 data set (14) was reprocessed using XDS (33) with cross-correlation to determine the diffraction limit (34) to a resolution of 2.9 Å. Structures were refined with PHENIX (36), manually built with Coot, and validated with MolProbity (22).

### Modeling

We used the MODELLER version 9.12 loop modeling protocol (21) to build and refine the eight missing residues between the prodomain and GF. Crystallographically defined regions were kept fixed. Ten loop models were built for each of the two connections, assessed based on the DOPE score (37), and validated using MolProbity (22).

## Author contributions

T. A. S. and B. Z. designed the experiments and wrote the manuscript. B. Z. carried out the biochemical studies and crystallization. X. D. performed data collection. B. Z., S. X., and X. D. performed structure determination. C. L. participated in the experiment design and data analysis.

## Supplementary Material

Supplemental Data

## Footnotes

3 The abbreviations used are:

GF: growth factor
GARP: glycoprotein-A repetitions predominant protein
PDB: Protein Data Bank
RMSD: root mean square deviation
PC: proprotein convertase.
