# Supplementary material for: Prodomain–growth factor swapping in the structure of pro-TGF-β1
Source: J Biol Chem. 2017 Nov 5;293(5):1579–89. doi: 10.1074/jbc.M117.809657 (PMC5798290; doi:10.1074/jbc.M117.809657)
Supplement: Supplemental Data [file supp_293_5_1579__index.html]

Prodomain-Growth Factor Swapping in the Structure of pro-TGF-β1 — Prodomain–growth factor swapping in the structure of pro-TGF-β1 — Prodomain–growth factor swapping in pro-TGF-β1 — Supplemental Data 

# Prodomain–growth factor swapping in the structure of pro-TGF-β1

## Supplemental Data

- Supplemental Figure 1 (.pdf, 2.3 MB) - Figure S1. Omit map showing the electron density of the connectivity region.
