# Supplementary material for: Prodomain–growth factor swapping in the structure of pro-TGF-β1
Source: J Biol Chem. 2017 Nov 5;293(5):1579–89. doi: 10.1074/jbc.M117.809657 (PMC5798290; doi:10.1074/jbc.M117.809657)
Supplement: Supplemental Data [file 10.1074_M117.809657_jbc.M117.809657-1.pdf]

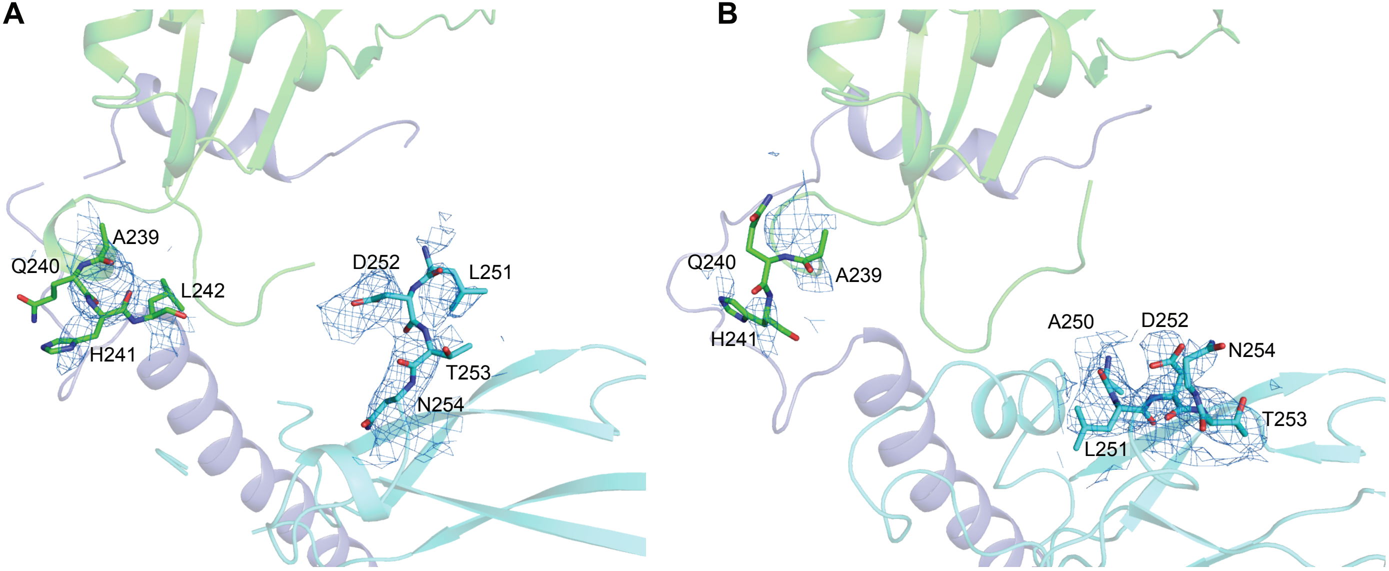

Figure S1. Omit map showing the electron density of the connectivity region. Simulated annealing composite omit 2Fo-Fc maps were calculated using Phenix. Electron density contoured at 1 σ is shown as blue mesh for the connectivity region between the prodomain arm domain and the growth-factor in the crystal structures of the uncleaved R249A PC mutant pro-TGF-β1 (A) and the cleaved WT pro-TGF-β1 (B). The arm domain is green, the straitjacket is lightblue, and the growth factor is cyan. Residues 239-242 of the prodomain and 251-254 for the growth-factor are shown as sticks.
